# Immunoliposome-PCR: a generic ultrasensitive quantitative antigen detection system

**DOI:** 10.1186/1477-3155-10-26

**Published:** 2012-06-22

**Authors:** Junkun He, David L Evers, Timothy J O’Leary, Jeffrey T Mason

**Affiliations:** 1 Biomedical Laboratory Research and Development Service, Veterans Health Administration, Washington, DC, USA; 2 Armed Forces Institute of Pathology, Rockville, MD, USA; 3 VA Medical Center, Research Building, Room GF-147, 50 Irving Street, N.W, Washington, DC, USA, 20422

## Abstract

**Background:**

The accurate quantification of antigens at low concentrations over a wide dynamic range is needed for identifying biomarkers associated with disease and detecting protein interactions in high-throughput microarrays used in proteomics. Here we report the development of an ultrasensitive quantitative assay format called immunoliposome polymerase chain reaction (ILPCR) that fulfills these requirements. This method uses a liposome, with reporter DNA encapsulated inside and biotin-labeled polyethylene glycol (PEG) phospholipid conjugates incorporated into the outer surface of the liposome, as a detection reagent. The antigenic target is immobilized in the well of a microplate by a capture antibody and the liposome detection reagent is then coupled to a biotin-labeled second antibody through a NeutrAvidin bridge. The liposome is ruptured to release the reporter DNA, which serves as a surrogate to quantify the protein target using real-time PCR.

**Results:**

A liposome detection reagent was prepared, which consisted of a population of liposomes ~120 nm in diameter with each liposome possessing ~800 accessible biotin receptors and ~220 encapsulated reporters. This liposome detection reagent was used in an assay to quantify the concentration of carcinoembryonic antigen (CEA) in human serum. This ILPCR assay exhibited a linear dose–response curve from 10^-10^ M to 10^-16^ M CEA. Within this range the assay coefficient of variance was <6 % for repeatability and <2 % for reproducibility. The assay detection limit was 13 fg/mL, which is 1,500-times more sensitive than current clinical assays for CEA. An ILPCR assay to quantify HIV-1 p24 core protein in buffer was also developed.

**Conclusions:**

The ILPCR assay has several advantages over other immuno-PCR methods. The reporter DNA and biotin-labeled PEG phospholipids spontaneously incorporate into the liposomes as they form, simplifying preparation of the detection reagent. Encapsulation of the reporter inside the liposomes allows nonspecific DNA in the assay medium to be degraded with DNase I prior to quantification of the encapsulated reporter by PCR, which reduces false-positive results and improves quantitative accuracy. The ability to encapsulate multiple reporters per liposome also helps overcome the effect of polymerase inhibitors present in biological specimens. Finally, the biotin-labeled liposome detection reagent can be coupled through a NeutrAvidin bridge to a multitude of biotin-labeled probes, making ILPCR a highly generic assay system.

## Background

The ability to accurately quantify specific antigens at low concentrations over a wide dynamic range is important in clinical medicine and many fields within the life sciences [1-4]. Advances in instrumentation and miniaturization are placing ever greater demands on assay technology, frequently requiring the detection of proteins at levels well below 1 picomolar and over a dynamic range as high as 10^6^. Examples include the detection of proteins in microgram tissue specimens isolated by laser capture microdissection [5] and the detection of proteins in nanoliter sample volumes used in high-throughput proteomic microarrays [6]. Conventional enzyme-linked immunosorbent assay (ELISA) methods [7] are incapable of accurately quantifying proteins over a wide dynamic range at this level of sensitivity. Currently, the only immunoassay method capable of fulfilling these criteria is immuno-PCR (IPCR). IPCR, first described by Cantor in 1992 [8], combines the specificity of antibody–protein binding with powerful polymerase-mediated nucleic acid amplification methods. A variety of IPCR assay formats have been introduced, which differ in the method used to couple the nucleic acid reporter to the antibody, the technique used for nucleic acid amplification, or the method used to detect the amplified nucleic acid reporters [9]. Unfortunately, these IPCR formats have several disadvantages. For one, the most sensitive IPCR assays use covalently coupled reporter DNA–antibody conjugates [9,10]. The preparation and purification of these conjugates requires expertise in protein conjugation chemistry, is time-consuming, and can result in low yields of the conjugate [11]. Second, in most IPCR assay formats there are no more than a few nucleic acid reporters coupled to each antibody, which makes detection of low copy number targets difficult in many specimens due to matrix effects, including the presence of polymerase inhibitors. Third, and most importantly, in all current IPCR methods the nucleic acid reporter of the conjugate is exposed to the assay solution, rendering it indistinguishable from nonspecific reporters that can arise from incomplete purification of the conjugates and inadvertent contamination during the IPCR assay procedure. This nonspecific reporter contamination is the source of the high and variable background signals that are common in the negative controls of IPCR assays [9,12-14]. Thus, IPCR is elegant in concept, but has proven frustratingly difficult in implementation.

We previously described an ultrasensitive immunoassay for detecting certain biological toxins that used liposomes with encapsulated DNA reporters, and ganglioside receptors embedded in the bilayer, as detection reagents [15]. Although this Liposome Polymerase Chain Reaction (LPCR) assay was highly sensitive, the use of gangliosides as receptors restricted the assay to the detection of a limited number of biological toxins.

**Figure 1:**
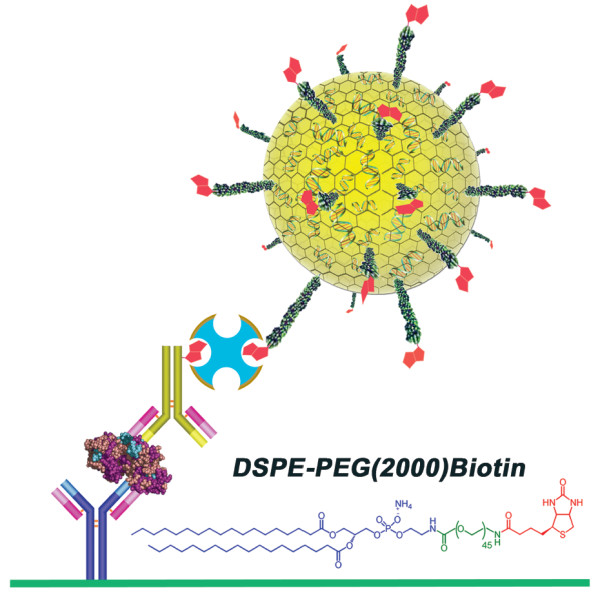
**Drawing depicting the ILPCR assay format.** The antigen (purple, brown, and blue) is bound by an immobilized capture antibody (blue and purple) and a biotinylated secondary antibody (green and purple). The liposome detection reagent (yellow) is coupled to the biotinylated secondary antibody through a NeutrAvidin bridge (aqua and brown). The biotin-labeled PEG phospholipid conjugates are pictured as PEG polymers (dark green) terminating in biotin molecules (red) with the phospholipid component (not visible) embedded in the outermost bilayer leaflet of the liposome. Encapsulated DNA reporters (green with red bars) can be seen inside the liposome. Shown at the bottom right is the biotin-labeled PEG phospholipid conjugate used to prepare the detection liposomes: 1,2-distearoyl-*sn*-glycero-3-phosphoethanolamine-*N*-[biotinyl (polyethylene glycol) 2000] ammonium salt. The phospholipid moiety is depicted in blue, the PEG (2000) polymer moiety in green, and the biotin moiety in red.

Here we report the development of a generic ultrasensitive quantitative antigen detection format called Immunoliposome Polymerase Chain reaction (ILPCR), which has been designed to overcome the disadvantages of IPCR and the limited applicability of our previous LPCR assay format. The term antigen is used in a broad sense to indicate any analyte for which antibodies are available. This includes antibodies themselves, which are important clinical biomarkers of disease. The ILPCR method (depicted in Figure 1) is demonstrated with an assay for carcinoembryonic antigen (CEA) in human serum. The detection reagent is a liposome (a hollow closed-shell nanosphere composed of a phospholipid bilayer) with reporter DNA encapsulated inside and biotin-labeled polyethylene glycol (PEG) phospholipid conjugates (Figure 1) incorporated into the outer bilayer leaflet. The biotin-PEG phospholipids serve as NeutrAvidin binding sites. The ILPCR assay follows a conventional ELISA format in which the target is immobilized inside a microplate well by a capture antibody followed by the addition of a biotinylated secondary antibody. The biotin-labeled liposome detection reagent is then coupled to the secondary antibody through a NeutrAvidin bridge. Any nonspecific DNA located outside the liposomes is degraded by treatment with deoxyribonuclease I (DNase I), followed by inactivation of the nuclease by heat. Throughout this process, the reporter DNA inside the liposomes is protected because DNase I cannot permeate the bilayer. The liposomes are then lysed with detergent to release the specifically bound encapsulated reporter DNA, which is detected by real-time quantitative PCR (qPCR). The ILPCR assay for CEA yielded a limit of detection (*LOD*) of 10^-16^ M, a dynamic range of 10^6^, and coefficient of variance (*CV*) values of <6 % for repeatability and <2 % for reproducibility. We also report the results of an ILPCR assay for p24, the core protein of human immunodeficiency virus-type1 (HIV-1), in buffer. More generally, the use of a NeutrAvidin bridge to couple the biotin-labeled immunoliposome to the secondary antibody makes ILPCR a generic ultrasensitive quantitative antigen detection system for the specific detection of a wide range of biomolecules.

## Methods

### Reagents and materials

DEAE-Sepharose CL-6B, bovine pancreatic DNase I, cholesterol, polyethylene glycol bisphenol A epichlorohydrin copolymer (PEG copolymer), bovine serum albumin (BSA) fraction V (RIA grade), phosphate buffered saline (PBS) tablets, and Triton X-100 (ultra grade) were obtained from Sigma-Aldrich (St. Louis, MO). Absolute ethanol was purchased from Pharmco-AAPER (Brookfield, IL). The phospholipids 1,2-distearoyl-*sn*-glycero-3-phosphocholine (DSPC); 1,2-distearoyl-*sn*-glycero-3-phosphoethanolamine-*N*-[biotinyl(polyethylene glycol)2000] ammonium salt [DSPE-PEG(2000)Biotin]; 1,2-distearoyl-*sn*-glycero-3-phosphoethanolamine-*N*-[methoxy(polyethylene glycol)2000] ammonium salt [DSPE-mPEG(2000)]; 1,2-dioleoyl-3-dimethylammoniumpropane (DODAP); and lissamine rhodamine B-1,2-dihexadecyl-*sn*-glycero-3-phosphoethanolamine triethylammonium salt (DHPE-rhodamine) were obtained from Avanti Polar Lipids (Alabaster, AL). NeutrAvidin, casein, other microplate blocking reagents, and the biotin quantitation kit were purchased from Pierce Biotechnology (Rockford, IL). Antibody coating solution was purchased from Kirkegaard and Perry Labs (Gaithersburg, MD). Corning high-binding microtiter plates (96-well) and SpectraPor Disposo Dialyzers with a molecular weight cut-off (WMCO) of 2,000 Da were obtained from Thermo-Fisher Scientific (Pittsburgh, PA). Polycarbonate membranes (13 mm diameter) were purchased from Costar Corporation (Cambridge, MA). Anti-CEA capture antibody, biotin-labeled anti-CEA secondary antibody, and recombinant human CEA antigen were obtained from US Biological (Swampscott, MA). Anti-p24 capture antibody, biotin-labeled anti-p24 secondary antibody, and recombinant p24 antigen were obtained from Abcam (Cambridge, MA). Primers were purchased from Integrated DNA Technologies (Coralville, IA). TaqMan universal PCR Master Mix, AmpliTaq Gold, and the Taqman probes for qPCR were purchased from Applied Biosystems (Foster City, CA). The DNA intercalating fluorescent dye TO-PRO-1 was purchased from Invitrogen (Carlsbad, CA). General reagents, solvents, and laboratory supplies were obtained from Sigma-Aldrich or Thermo-Fisher Scientific. All work involving human serum was carried out using universal precautions. The use of de-identified human serum in this study was ruled exempt by the Institutional Review Board of the Armed Forces Institute of Pathology (AFIP protocol approval code: UBUC).

### Instruments

Optical absorbance measurements were recorded with a Beckman model DU-640 UV–vis spectrophotometer (Fullerton, CA). Fluorescence measurements were made with a SpectraMax M5 microplate reader from Molecular Devices (Sunnyvale, CA). Liposome sizing by extrusion through polycarbonate membranes was carried out with a temperature-jacketed Thermobarrel Extruder from Lipex Biomembranes (Vancouver, Canada). Temperature control during extrusion was achieved by connecting a circulating water bath to the water jacket of the extruder. Dynamic light scattering measurements were made with a Nicomp model 370 autocorrelation light scattering spectrometer from Particle Sizing Systems (Santa Barbara, CA). Microtiter plates were washed using a BioTek model ELx405 automated plate washer (Winooski, VT). Real-time PCR measurements were carried out using an ABI model 7500 Real Time PCR System (Applied Biosystems Incorporated) unless otherwise noted in the text.

### Preparation of reporter DNA

The reporter DNA that is encapsulated inside the liposomes serves only as a PCR amplification template for the detection and quantification of the corresponding analyte, thus the specific sequence is not critical [16]. In general, the template should be <100 base-pairs in length to maximize encapsulation into the liposomes and consist of a sequence not likely to be found in the samples being analyzed. For the CEA assay an 84-base segment derived from the human β_2_-microglobin transcript was used. This segment spans an intron and thus is unlikely to be present in human serum. The reporter was prepared by cloning β_2_-microglobin cDNA, prepared and amplified from HeLa cell RNA, into a pCR2.1-TOPO T/A plasmid vector, which was used to transform On-Shot *E*. *Coli* (Invitrogen). A detailed description of reporter preparation using this method [16] is given in additional file 1: Supplementary information, under the section entitled “Preparation of DNA reporters”. The reporters used in the assay control studies, which were derived from the Norway rat glutamate receptor-interacting protein 1 (GRIP1) and tobacco mosaic virus (TMV) 126 kDa coat protein sequences, were purchased commercially from Integrated DNA Technologies. Detailed information on all three reporters used in this study, including their sequences and corresponding primers and probes are given in additional file 1: Supplementary information, under the section entitled “Reporters, primers, and probes”.

### Preparation of the liposome detection reagent

Liposomes were prepared by mixing chloroform solutions of DSPC (24.5 mol %), cholesterol (45 mol %), DODAP (25 mol %), DSPE-mPEG(2000) (4.75 mol-%), DSPE-PEG(2000)Biotin (0.25 mol %), and DHPE-rhodamine (0.5 mol %). The solvent containing the lipid mixture (25 mg total lipid) was evaporated by drying under a stream of N_2,_ and then under high vacuum for at least 4 h. The dried lipid film was hydrated in 1 mL of 300 mM citrate buffer, pH 4, by vortexing the suspension at 70°C. The resulting multilamellar vesicles were then subjected to five freeze/thaw cycles using liquid nitrogen and a water bath set to 70°C. The liposomes were extruded 10 times through two stacked 0.1-micron polycarbonate membranes at 70°C using a vesicle extruder, which led to the formation of unilamellar liposomes ~100 nm in diameter. Ethanol was then slowly added to the rapidly vortexed liposome suspension until the final ethanol concentration was 40 % by volume. The reporter DNA (300 μg) was added to the liposomes, which were incubated at 40°C for 1 h and then dialyzed against 2 L of the citrate buffer using a 2,000 Da MWCO Disposo Dialyzer. The preparation was then dialyzed against 2 L of 20 mM HEPES buffer, 145 mM NaCl, pH 7.5. Unencapsulated reporter DNA was removed by ion-exchange gel filtration on DEAE-Sepharose CL-6B (0.5 ml of gel/mg total lipid) using the HEPES buffer [17]. False-negative control liposomes were prepared as described above, but with 5 mol% DSPE-mPEG(2000) and no DSPE-PEG(2000)Biotin. The 81 base-pair reporter derived from the TMV 126 kDa coat protein sequence was encapsulated into the liposomes.

### Determination of total lipid and total reporter concentration

DHPE-rhodamine (0.5 mol%) was included in the liposomes to facilitate the determination of lipid concentration. A 25-μL aliquot of the liposome solution was added to a test tube along with 1.5 mL of methanol and 20 μL of 0.1 N NaOH [18]. A blank was similarly prepared using PBS. The absorbance of the solution was read at 560 nm (A_560_) in a 1-cm path-length cell after zeroing the spectrophotometer against the blank. The total lipid concentration of the liposome solution was then calculated as A_560_ x 130 μmol/mL (A_560_ x 88 mg/mL). A 25-μl aliquot of the liposome solution was combined with 350 μL of 1 M NaCl and 1.125 mL of chloroform/methanol (2:1, v/v). A blank was similarly prepared using 25 μL of PBS. The solutions were vortexed and allowed to stand for 10 min then the upper aqueous phase of each solution was removed by careful pipetting. The absorbance of the reporter DNA was then read at 260 nm (A_260_) in a 1-cm path-length cell after zeroing the spectrophotometer against the blank. The β_2_-microglobin reporter concentration was then calculated as A_260_ x 55.8 nmol/mL (A_260_ x 1,458 μg/mL).

### Determination of encapsulated reporter

The relative distribution of reporter DNA in free solution versus that encapsulated inside the liposomes was determined with a fluorescence assay using the DNA intercalating dye TO-PRO-1. The liposome detection reagent (25 μL) was added to 2 mL of 20 mM HEPES, 145 mM NaCl, pH 7.5, along with 1 μL of 1 mM TO-PRO-1 in DMSO [19]. A blank was similarly prepared using 25 μL of PBS. The fluorescence intensity (I_1_) was then measured at 532 nm using an excitation of 514 nm. The liposomes were then ruptured by adding 20 μL of 100 mM Triton X-100 followed by incubation at 37°C for 15 min. The fluorescence intensity was measured again as described above (I_2_). The ratio of the two fluorescence measurements (after correcting for dilution) yields the fraction of free (I_1_/I_2_) and encapsulated [1-(I_1_/I_2_)] reporter. The actual DNA concentrations were determined by combining the TO-PRO-1 distribution with the total DNA concentration measured as described above. The encapsulated DNA was then normalized to the total lipid concentration to yield the number of mmol of encapsulated reporter per mol total lipid.

### Determination of liposome size

The hydrodynamic diameter of the liposomes was determined at 24°C by dynamic light scattering using a ~1:500 dilution of the liposome detection reagent in PBS. Scattered light was measured at a 90° angle using an external 75 mW argon-ion laser operating at 488 nm. A 6.7 μsec channel width and an intensity of 400 kHz were used for data collection. A refractive index of 1.33 and a viscosity of 1.05 cP were used for PBS at 24°C. Data was accumulated for 15–20 min, which was sufficient to yield a correlation function decay of 2.3 ensuring accurate sizing of the liposomes. The autocorrelation function was fit to a Gaussian distribution using number-weighted averaging corrected for hollow particles. The resulting particle distribution was plotted as a bar graph of the relative number of liposomes versus size plotted on a logarithmic scale.

### Estimation of available biotin

The DSPE-PEG(2000)Biotin exposed on the outer surface of the liposome detection reagent and thus available for binding to NeutrAvidin was determined using a 4'-hydroxyazobenzene-2-carboxylic acid-avidin displacement spectrophotometric assay kit from Pierce Biotechnology. The biotin concentration was measured by the decrease in absorption at 500 nm following the addition of the liposome detection reagent to the assay solution. Liposomes prepared with DSPE-mPEG(2000) in place of the biotin analogue were used as a blank to zero the spectrophotometer. The exposed DSPE-PEG(2000)Biotin was then normalized to the total lipid concentration to yield the number of mmol of exposed biotin per mol total lipid.

### Buffers used in the ILPCR assay

The following buffers were used in the ILPCR assay:

*Coating buffer:* 50 mM bicarbonate, pH 9.6

*Buffer A (PBST):* 2 mM imidazole/0.02 % (w/v) Tween-20 in PBS, pH 7.4

*Buffer B (PBS):* 10 mM PBS, pH 7.4

*Buffer C:* 1 % (w/v) BSA in PBST, pH 7.4

*Buffer D:* 1 % (w/v) casein in 10 mM PBS, pH 7.4

*Buffer E:* 1 % (w/v) PEG copolymer in deionized water

*Digestion buffer:* 10 mM CaCl_2_, 10 mM MgCl_2_, 20 mM HEPES, pH 7.8

*Lysis buffer:* 10 mM Triton X-100 in 10 mM borate, pH 9.0

### ILPCR assay for CEA in human serum

A volume of 100 μL of capture antibody (1.5 μg/mL) in coating buffer was added to the inner 60 wells of a 96-well microtiter plate. The plate was then sealed and incubated overnight at 4°C. The coating buffer was aspirated, and the plate wells were washed twice with 300 μL of *buffer A*. Each plate well received 100 μL of serially diluted recombinant CEA in human serum (concentration range: 10^-8^ to 10^-18^ M CEA) or *buffer C* only (blank and no-template controls), with each of these prepared in triplicate. Each serum sample was diluted with PBS (1:10) prior to its addition to the plate well. The plate was sealed and allowed to incubate at 37°C for 1 h after which the solutions were aspirated and the wells were washed twice with *buffer A*. The wells were then blocked with casein by adding 300 μL of *buffer D* and incubating the plate at room temperature (RT) for 1 h. The wells were aspirated and washed twice with 300 μL of *buffer A*. A volume of 100 μL of biotinylated secondary antibody (1 μg/mL) in *buffer* C was added to each well, and the plate was allowed to incubate at 37°C for 1 h. The solution was then aspirated, and the wells were washed twice with 300 μL of *buffer A*. A volume of 100 μL of NeutrAvidin (2 μg/mL) in *buffer B* was added to each well and the plate was incubated at 37°C for 1 h. The solution was aspirated and the wells were washed twice with 300 μL of *buffer B*. The plate wells were then blocked a second time with casein and washed as described above. A volume of 100 μL of liposome detection reagent at a concentration of 100 nM (0.1 nmol total lipid/mL) in *buffer E* was added to each well (except the 3 wells serving as the no-template control), and the plate was incubated at RT for 1 h. The wells were then washed 5 times with 300 μL of *buffer B*. Each well then received 100 μL of DNase I (10 U/well) in *digestion buffer* to degrade any unencapsulated DNA. The digestion was carried out at 37°C for 20 min, and the DNase I was then inactivated by heating the plate at 80°C for 10 min. The wells were washed 5 times with 300 μL of *buffer B*. Finally, the liposome detection reagent was lysed by the addition of 100 μL of *lysis buffer* per well, followed by incubation at RT for 20 min on a plate shaker at 600 rpm.

### ILPCR assay for p24 in buffer

The assay for p24 was carried out exactly as described above for CEA. Each plate well received 100 μL of serially diluted recombinant p24 in PBS (concentration range: 10^-7^ to 10^-17^ M p24) or *buffer C* only (blank and no-template controls), with each of these prepared in triplicate. Deactivation of DNase I was carried out by heating or by an alternate method as described under Results and Discussion.

### Quantitative PCR

Following lysis of the liposomes, a 1-μL aliquot from each microtiter plate well was added to 12.5 μL of 2x TaqMan Universal PCR Master Mix. Each PCR tube then received 1 μL of forward and reverse primers (15 μM each), and 1 μL of the probe (5 μM). Water was added to bring the reaction volume to 25 μL. PCR was preceded by a 2-min UNG incubation step at 50°C and a 10-min Ampli Taq Gold activation step at 95°C. Forty cycles of PCR were then performed, where each cycle consisted of a 15-sec denaturation step at 95°C and a 1-min annealing/extension step at 60°C. All primer and probe design was performed using “Taqman Probe & Primer Design” software (Applied Biosystems Incorporated). The primers used in the real-time PCR assay for β_2_-microglobin were:

β_2_M-246F (forward): 

β_2_M-330R (reverse): 

The fluorescent probe used for β_2_-microglobin was:

For complete information on the β_2_-microglobin reporter see additional file 1: Supplementary information, under the section entitled “Reporters, primers, and probes”.

## Results and discussion

### Preparation of the liposome detection reagent

The method used to prepare the liposome detection reagent was a modification of the approach pioneered by Maurer et al. [17] for the encapsulation of anti-sense RNA into liposomes using cationic lipids. A total PEG-phospholipid concentration of at least 5 mol% was necessary to promote the extension of the PEG polymers into the surrounding aqueous phase. At lower concentrations the PEG polymers were inaccessible for binding, presumably due to self-aggregation [20]. PEG-phospholipid concentrations greater than 10 mol% destabilized the bilayer leading to leakage of the reporter and a reduction in the shelf-life of the detection reagent. The mol% DSPE-PEG(2000)Biotin incorporated in the liposomes was varied between 0.1 and 1.5 mol% to determine the optimal concentration for binding to NeutrAvidin. Binding avidity improved up to 0.5 mol%, but did not improve with higher concentrations of DSPE-PEG(2000)Biotin.

In order to ensure proper extrusion of the liposomes the following precautions were observed. The polycarbonate filters were hydrated in buffer before adding them to the extruder and the liposome solution was added to the bottom of the extruder barrel using a plastic pipette bulb in order to avoid generating an air space above the filters. If the extrusion was unacceptably slow at a nitrogen pressure of 600 psi, 5–10 extrusions through a 0.2-micron polycarbonate filter were performed prior to extrusion using the 0.1-micron filters. The gel column purification method was satisfactory for most cases as any contaminating non-encapsulated DNA was digested during the ILPCR assay. For characterizing the liposomes a more rigorous purification can be achieved by digesting the non-encapsulated DNA prior to the column purification step using the method of Monnard et al. [21], which is described in additional file 1: Supplementary information, under the section entitled “Pre-column nuclease digestion of reporters”.

The detection liposome preparation method proved to be highly reproducible. Four preparations of the detection reagent (using different batches of lipid and reporter) adjusted to a total lipid concentration of 100 nM yielded *Ct* values between 15.81 and 16.94 with a standard deviation of 0.79 following lysis with Triton X-100 and analysis by qPCR. This yielded a coefficient of variation (*CV*) of 6 % for the reproducibility of the liposome detection reagent (Table 1). The key step to ensuring this level of reproducibility was to add the ethanol and DNA slowly while rapidly vortexing the lipid solution in order to prevent the formation of large aggregates. Each solution was added over the course of ~1 minute using a syringe with a small-bore (18–20 gauge) needle. Also, the solutions were heated to 70°C before mixing.

**Table 1:** **Parameters of the liposome detection reagent**^**a)**^

**Parameter**	**Value**
Hydrodynamic diameter ^b)^	117 ± 20 nm
Exposed biotin/lipid molar ratio ^c)^	5.1 ± 0.2 mmol/mol
Reporter DNA/lipid molar ratio ^d)^	2.1 ± 0.4 mmol/mol
*CV* of liposome reagent reproducibility ^e)^	6 %
Liposome reagent stability ^f)^	1.5 years at 4 °C

^a)^ The parameters were determined using measurements from 4 replicate preparations of the liposome detection reagent.

^b)^ The hydrodynamic diameter of the liposomes was determined by dynamic light scattering using a number-weighted Gaussian size distribution.

^c)^ The average number of biotin molecules exposed on the surface of the liposomes was estimated using a 4'-hydroxyazobenzene-2-carboxylic acid-avidin displacement quantification assay and the total lipid concentration. The total lipid concentration was determined from the absorbance of DHPE-rhodamine.

^d)^ The ratio of reporter DNA to total lipid, where the reporter concentration was measured by its absorbance at 260 nm.

^e)^ The *CV* for the reproducibility of the liposome detection reagent preparation was determined from the four replicate preparations by measuring the *Ct* value associated with equal concentrations of total lipid.

^f)^ The liposome detection reagent stability was defined as the length of time the liposomes could be stored at 4°C without observing a reduction in either the *LOD* or dynamic range when performing the CEA assay. See Table 2 for the *LOD* and dynamic range of the ILPCR assay for CEA in human serum.

### Characterization of the liposome detection reagent

**Figure 2:**
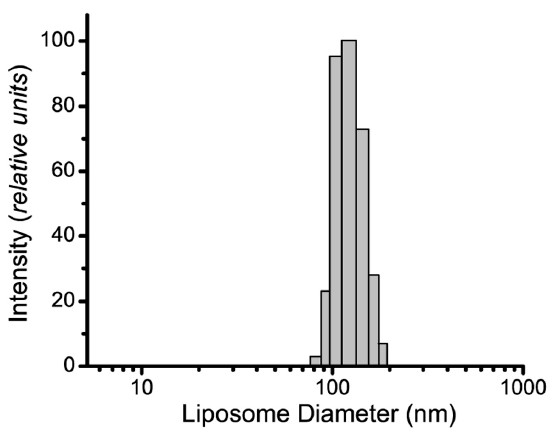
**Size distribution of the detection liposomes.** Dynamic light scattering was used to determine the number-weighted distribution of liposome sizes in the detection reagent preparation. The range of liposome diameters spanned 75–180 nm, with ~75 % of the liposomes having diameters between 100 and 150 nm. The distribution was monodisperse, with a mean diameter of 117 nm and a standard deviation of ±20 nm.

Dynamic light scattering was used to determine the number-weighted distribution of liposome sizes in the detection reagent preparation (Table 1, Figure 2). The liposomes revealed a monodisperse distribution of vesicle sizes with a mean liposome diameter of 117 nm and a standard deviation of ±20 nm. The range of liposome diameters spanned 75–180 nm, with ~75 % of the liposomes having diameters between 100 and 150 nm. No liposome aggregates >500 nm in diameter or smaller structures <10-nm in diameter, consistent with PEG phospholipid micelles, were detected. The percentage of reporter DNA encapsulated inside the detection liposomes was determined by fluorometric assay using the DNA intercalating dye TO-PRO-1 [19]. At least 96 % of the reporter was encapsulated inside the liposomes following the column purification step. This value increased to >99 % if the DNA was hydrolyzed prior to column purification [21].

We typically used 300 μg of DNA when preparing the detection liposomes. This resulted in ~2 mmol of encapsulated reporter per mol of total lipid (Table 1), which represented an encapsulation efficiency of 52 %. Analysis of the liposomes by ^31^P NMR in the presence and absence of manganese chloride, a shift reagent [22], indicated that both unilamellar and multilamellar liposomes were present in the preparation (data not shown). Consequently, it was difficult to estimate the quantity of encapsulated DNA per liposome. An approximation was obtained by assuming that the liposomes were unilamellar with a diameter of 117 nm, which yields a lower-limit estimate of 220 reporters per liposome [23]. The quantity of DSPE-PEG(2000)Biotin on the outer surface of the liposomes available for binding to NeutrAvidin was estimated using a 4'-hydroxyazobenzene-2-carboxylic acid-avidin displacement quantification assay. This analysis yielded a value of ~5 mmol of surface biotin per mol of total lipid (Table 1). This yielded a lower-limit estimate of 800 biotin binding sites per liposome by using the unilamellar liposome approximation discussed above.

### Shelf-life of the liposome detection reagent

The stability (shelf-life) of the liposome detection reagent was defined as the length of time the liposomes could be stored at 4°C without observing a reduction in either the *LOD* or dynamic range when performing the CEA assay. Given this criteria, the shelf-life was ~1.5 years (Table 1). The shelf-life was reduced to about 1 month when the liposomes were stored at room temperature. The liposomes were assessed periodically with regard to their DNA and DSPE-PEG(2000)Biotin content and their aggregation state. The liposomes lost 18 % of their encapsulated DNA and 2 % of their biotin content over a period of one year. The released reporter did not interfere with the ILPCR assay as it is digested by the DNase I treatment during the assay. The reduced level of encapsulated reporter was compensated by performing a standard curve with each ILPCR assay. About 5 % of the liposomes had aggregated over a period of one year. Liposome aggregation was defined as the presence of lipidic particles >500 nm in diameter when the liposomes were analyzed by dynamic light scattering.

The long shelf-life of the liposome detection reagent was likely due to the prevention of destabilizing bilayer contacts by the PEG polymers [24]. Another effect of the PEG polymers was to render the liposomes approximately equal in density to the HEPES buffer in which they were suspended, facilitating the accurate pipetting of the liposome solution. This is frequently a shortcoming of other nanoparticle-based assays. Also, unlike gold, silver, or magnetic particle-based assays, the liposomes used in the ILPCR assay are nontoxic and biodegradable “green” nanotechnology. Finally, the liposome detection reagent was easy and inexpensive to prepare and, combined with its long shelf-life, the assay cost associated with using the liposome detection reagent is considerably less than for IPCR or nanoparticle-based assay methods.

### Optimization of ILPCR assay performance

The following components were evaluated to determine their effect on ILPCR assay performance: the type of microtiter plate, the capture and biotin-labeled secondary antibodies, the antibody coating solution, the blocking reagents, the incubation and wash buffers, the type of avidin derivative used, and the properties of the liposome detection reagent. Once the optimal components were identified the following parameters were evaluated to determine their effect on ILPCR assay performance: the concentration of all assay components, the number of blocking steps, all incubation times and temperatures, the number of wash steps and cycles performed, and the ionic strength of the wash buffers. The final concentrations of the biotin-labeled secondary antibody, the NeutrAvidin, and the liposome detection reagent were optimized by the iterative method described by Wu et al. [25] for streptavidin-based IPCR assays.

Corning high-binding EIA/RIA grade polystyrene 96-well microtiter plates with flat bottoms yielded the most consistent results and the highest sensitivity as did immobilization of capture antibody in 50 mM sodium bicarbonate buffer, pH 9.6, using 0.15 μg of antibody per well. Streptavidin, NeutrAvidin, and Avidin were evaluated as the bridge between the biotin-labeled secondary antibodies and liposomes, with NeutrAvidin yielding the lowest background while maintaining high sensitivity. A wash buffer of 2 mM imidazole/0.02 % (w/v) Tween-20 in PBS, pH 7.4, proved optimal for all assay steps other than those involving the detection liposomes, where the presence of detergent was avoided to prevent disruption of the liposomes.

Degradation of non-encapsulated DNA was achieved by the addition of 10U of DNase I per plate well followed by incubation for 20 min at 37°C. These conditions were sufficient to hydrolyze the reporter encapsulated in 0.1 nmol of total lipid, which was ~10-times the amount added to each plate well. The amount of detergent added per plate well (100μL of 10 mM Triton X-100) was also sufficient to lyse this concentration of liposomes and quantitatively release the encapsulated reporter. The small quantity of Triton X-100 transferred to the PCR reaction mixture had no effect on qPCR assay performance.

Nonspecific protein binding was optimally blocked with 1 % (w/v) BSA (RIA grade) in PBST. The best blocking agent to minimize nonspecific binding of the liposome detection reagent was 1 % (w/v) casein in PBS. Optimal ILPCR assay performance was achieved by including two casein blocking steps, the first after the addition of the specimen (antigen) and the second after the addition of NeutrAvidin. Nonspecific liposome binding was also improved by diluting the liposomes in 1 % (w/v) PEG copolymer in PBS. This was not unexpected as PEG copolymers have been highly effective in blocking nonspecific binding in a variety of immunoassay formats [26,27]. The incorporation of phospholipid-PEG conjugates into the bilayer was a highly effective means to reduce liposome nonspecific binding. The PEG polymers served to limit the overall interaction of the liposome with its assay environment to that of the tips of the polymer chain, shielding the much larger hydrophilic surface of the liposome from destabilizing contacts with proteins and the plastic surface of the plate well [28]. Similar results were found in an immunoassay that used luminescent polystyrene beads covalently labeled with PEG polymers as a detection reagent [29].

**Figure 3:**
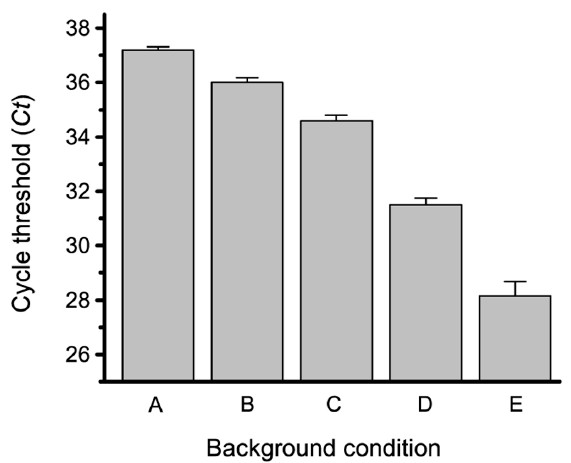
**Effect of different reagents on the background of the ILPCR assay.** Various combinations of the assay reagents were assessed for their effect on the non-specific background signal (noise) of the ILPCR assay. Column A: all assay reagents minus the liposome detection reagent. Column B: all assay reagents minus the antigen, the biotin-labeled secondary antibody, and the NeutrAvidin. Column C: all assay reagents minus the secondary antibody. Column D: all assay reagents minus the antigen, which represents the true assay blank. Column E: all assay reagents minus the antigen (as in D), but with no DNase I digestion step. Measurements were performed using a Bio-Rad model CFX96 real-time PCR system (Hercules, CA).

Various combinations of the assay components were assessed for their effect on the nonspecific background signal of the ILPCR assay with results shown in Figure 3. Column A was the result of an ILPCR assay that contained all assay components except the liposome detection reagent, which was equivalent to a no-template control (*Ct* = 37.2). Column B reflected the nonspecific binding of the liposome detection reagent as the only components present were the capture antibody and the two blocking agents, BSA and casein (*Ct* = 36.1). Column C was the result of an ILPCR assay that contained all of the assay components except the secondary antibody. It represented the contribution of NeutrAvidin to the background signal (*Ct* = 34.7). Column D was the results of an ILPCR assay with all of the assay components except the antigen (*Ct* = 31.6), which represented the true assay control (blank). Column E was a repeat of the control assay of column D, but with no DNase I digestion step (*Ct* = 28.2). The results of this study revealed that the nonspecific background signal of the ILPCR assay resulted from the cumulative effect of all of the assay components, with nonspecific binding of the biotin-labeled secondary antibody having the greatest effect. The study also revealed that the implementation of a DNA digestion step significantly reduced both the intensity and variability (standard deviation) of the nonspecific background signal.

### CEA and anti-CEA monoclonal antibodies

CEA, a heavily glycosylated protein with a molecular mass of 150 kDa, is a member of the CEA subfamily, which, in turn, is a member of the immunoglobulin gene superfamily [30,31]. CEA is anchored to the apical surface of epithelial cells through linkage to glycosyl phosphatidylinositol where it functions, principally, as an intercellular adhesion molecule. Soluble CEA detected in circulation is equivalent to the extracellular domain released from tumor cells by treatment with bacterial phosphatidylinositol-specific phospholipase C [32]. CEA is a nonspecific tumor marker as its expression is elevated in many epithelial tumors in addition to certain nonmalignant diseases, and it is also expressed in many normal tissues [33]. Clinically, CEA is used primarily as a serum marker for monitoring recurrence of colorectal carcinoma following surgical resection [34].

CEA consists of one *N*-terminal (Ig)V-like domain and six (Ig)C2-like domains, with a domain organization of: N-A1B1-A2B2-A3B3-C [35]. Monoclonal antibodies (mAbs) against CEA predominantly recognize protein epitopes and not the carbohydrate moiety [36]. Further, most anti-CEA mAbs recognize one of five non-interacting epitope groups, designated GOLD 1–5 [37] that are conformational rather than linear in nature [35]. The capture mAb used in the ILPCR assay is designated as clone 12-140-1 (IgG1) and recognizes a conformational epitope in the *N*-terminal region (Gold 5), of CEA [37] while the biotin-labeled secondary antibody, designated clone 12-140-10 (IgG1), recognizes a conformational epitope in the A1B1 region (Gold 4). Both mAbs have a *K* of 4 x10^-11^ M [38].

### ILPCR assay for CEA in human serum

A titration series was prepared by adding recombinant human CEA to CEA-negative human serum. The mean *Ct* value and the standard deviation of the calibration standards, the blank, and the controls were calculated using the three replicate measurements from the qPCR analysis. Controls were run for the lysis buffer, water, and PCR reaction mixture, including primers and probes, in addition to the no-template control. These controls should have *Ct* values >35. The three blanks should have a mean *Ct* value ≥30, with the preferred value being 31–32. A mean blank *Ct* value below 30 could indicate contamination of one of the reagents. A standard curve was constructed from the calibration standards by plotting the average *Ct* values versus the log of the antigen concentration. The linear region of the dose–response curve was identified by visual inspection and subjected to a linear regression analysis along with calculation of the 95 % confidence limits. The assay threshold, which was defined as the average *Ct* value of the blank minus three times the standard deviation of the blank [25], was then determined. This value defined the minimum detectable concentration (*MDC*) of the assay. The assay *LOD* is defined as the lowest concentration of analyte that is both within the linear region of the dose–response curve and below the assay threshold. For best results, the standards and controls should be run in the same biological matrix as the sample specimens being analyzed [16].

**Figure 4:**
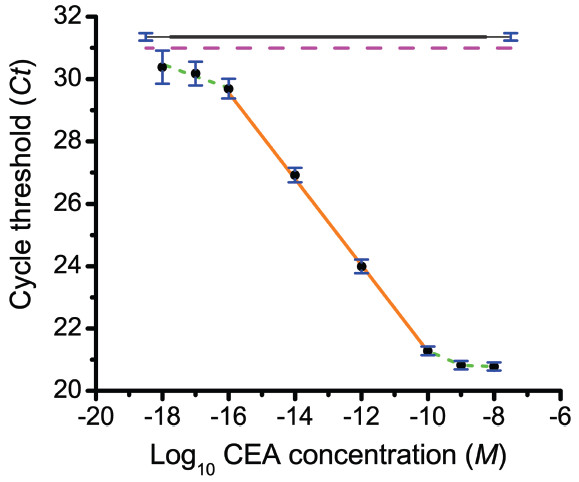
**Quantitative ILPCR dose–response curve for CEA added to CEA-negative human serum.** A 100-μl sample volume (antigen diluted in CEA-negative human serum) was used for all concentrations. The black circles are the average of three replicate *Ct* measurements over a concentration range of 10^-8^ to 10^-18^ M CEA; the standard deviation of the *Ct* values are shown as blue vertical bars. The orange line is the linear regression fit of the data from 10^-10^ to 10^-16^ M CEA. The green dotted lines depict the trend in the data outside the major linear region of the dose–response curve. The solid grey line is the average *Ct* value of the blank with the standard deviation shown in blue at each end of this line. The blank *Ct* value was plotted as a line rather than a single point for ease of visualization. The dashed magenta line is the detection threshold of the assay, which is defined as the average *Ct* value of the blank minus 3 times the standard deviation of the blank.

**Figure 5:**
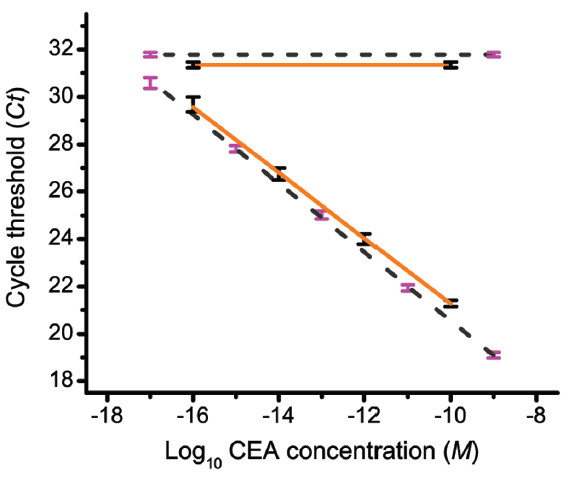
**The effect of the sample matrix on the performance of the ILPCR assay.** The solid orange lines with the accompanying data points and their standard deviations shown in blue are derived from Figure 4 for the ILPCR assay of CEA added to CEA-negative human serum. The dashed grey lines with the accompanying data points and their standard deviations shown in magenta represent an ILPCR assay for CEA added to PBS. Other parameters are as defined in Figure 4.

The result of this assay is shown in Figure 4, and the performance characteristics of the assay are given in Table 2. The linear region of the dose–response curve extended from 10^-10^ to 10^-16^ M (6 orders of magnitude). The *LOD* was 10^-16^ M, which corresponds to 13 fg/ml of CEA or 6,023 molecules (10 zeptomoles) of CEA in a 100-μL serum sample. This *LOD* was >1,500 times lower than the best clinically-approved ELISA or RIA tests for CEA [39-41], while IPCR assays for CEA in serum [42,43] reported an *LOD* ≥ 900,000 molecules and a dynamic range of 10^3^. The ILPCR assay results were independent of serum dilution, demonstrating the insensitivity of the assay to matrix effects. Also, the dose–response curve for CEA in human serum was almost identical to that for CEA in PBS as shown in Figure 5. In the range of 1,000 to 10,000 molecules, the assay precision yielded a minimal distinguishable difference of ~510 molecules (Table 2) based on three replicate measurements.

**Table 2:** **Parameters of the ILPCR assay for CEA in human serum**^**a)**^

**Parameter**	**Value**
*CV* of repeatability ^b)^	3 % (10^-10^ M) to 6 % (10^-16^ M)
*CV* of reproducibility ^b)^	0.7 % (10^-10^ M) to 1.8 % (10^-16^ M)
Linear correlation coefficient (*r*) ^c)^	0.998
Dynamic range	10^6^
Detection threshold (*Ct*) ^d)^	30.97
Limit of Detection (*LOD*) ^e)^	10^-16^ M (13 fg/ml), ~6,000 molecules
Precision at *LOD*^f)^	~500 molecules
Minimum Detectable Concentration (*MDC*) ^g)^	10^-17^ M (1.3 fg/ml), ~600 molecules
Sensitivity ^h)^	100 % (10^-15^ M), 87 % (10^-16^ M)
Specificity ^i)^	100 %

^a)^ All parameters were determined using measurements from a total of 11 assays (*n* = 11).

^b)^ Coefficient of variance (*CV*) values increase with decreasing CEA concentration likely due, in part, to the stochastic effects associated with measuring very low analyte concentrations in small sample volumes.

^c)^ The indicated value is the linear correlation coefficient resulting from the fit of the data from 10^-10^ to 10^-16^ M CEA of the dose–response curve shown in Figure 4.

^d)^ The detection threshold is a measure of the noise level of the assay and is defined as the average *Ct* of the blank (all assay components except the antigen) minus 3 times the standard deviation of the blank.

^e)^ The *LOD* is defined as the lowest concentration within the linear region of the dose–response curve that yields a *Ct* value ≤ the detection threshold. The *LOD* corresponds to a *Ct* value of 29.32, which is well below the detection threshold of the assay (*Ct* = 30.97).

^f)^ A measure of the assay precision at the *LOD* was estimated by determining the number of molecules in the 100-μL sample at the *LOD* (6,023) and the associated upper and lower 95 % confidence limits, which yielded values of 6,530 and 5,550 molecules, respectively.

^g)^ The *MDC* is the lowest CEA concentration that is ≤ the detection threshold as determined from the second linear region of the dose–response curve of Figure 4 (the green dashed line from 10^-16^ to 10^-18^ M CEA).

^h)^ The assay sensitivity (percentage of the assays yielding an *LOD* ≤ the indicated CEA concentration) based upon the eleven ILPCR assays performed.

^i)^ The assay specificity was determined using multiple samples derived from a single CEA-negative human serum reference specimen; therefore, the specificity must be taken as preliminary.

The dose–response curve in Figure 4 flattened abruptly at the high concentration end of the curve. This was likely due to the formation of a monolayer of liposomes over the surface of the plate well, preventing further binding of liposomes to immobilized antigen [18]. This suggested that higher concentrations of antigen may be measured by diluting either the concentration of antigen or the liposome detection reagent. In contrast, the low concentration end of the curve did not flatten, but instead displayed a second linear region with significantly reduced slope. This second linear region is characteristic of IPCR-based immunoassays [25,44], and it defines the *MDC* of the ILPCR assay, which was 10^-17^ M or ~ 600 molecules of CEA (1.3 fg/mL). At very low concentrations the precision was likely limted, in part, by the stochastic effects associated with measuring small sample volumes at low concentrations [45,46].

Of greater importance were the performance characteristics of the ILPCR assay over the concentration range of 10^-9^ M (nM) to 10^-15^ M (fM), which is the range most critical for high-sensitivity clinical assays. Within this range, the CEA assay displayed 100 % sensitivity and specificities of <5 % for repeatability and <2 % for reproducibility. These performance characteristics were achieved even when using different preparations of the liposome detection reagent and different lots of the antibodies, NeutrAvidin, casein, and microtiter plates. In Table 3 we compared the performance of the ILPCR assay to that of other published CEA assay formats. This was evaluated by conducting a literature search to identify all CEA assays that measured CEA in serum and reported at least an *LOD* and dynamic range. Eighteen assay formats were identified and the assay reporting the lowest *LOD* for CEA was listed in Table 3 for each of the 18 formats. ILPCR had an *LOD* 8-times lower and dynamic range 1,000-times greater than the next most sensitive assay.

**Table 3:** **Comparison of the dynamic range and** ***LOD*** **for different CEA assay formats**^**a)**^

**Assay format**	**Range (ng/mL)**	***LOD*** **(pg/mL)**^**b)**^	**Reference**
Radioimmunoassay	5 – 320	5,000	[41,60]
Chemiluminescence ^c)^	1 – 25	500	[61]
Quartz-crystal microbalance	2.5 – 55	500	[62]
Microarray fluorescence sensor ^d)^	0.16 – 9.4	400	[63]
Time-resolved fluoroimmunoassay ^e)^	1 – 560	280	[64]
Electrochemiluminescence ^f)^	0.21 –2,000	200	[65]
ICP Mass spectrometry ^g)^	15 – 250	140	[66]
Amperometric immunosensor ^h)^	0.2 – 160	60	[67]
Microchip electrophoresis ^i)^	0.06 – 8	46	[68]
ICP mass spectrometry (immunogold) ^j)^	0.07 – 1,000	30	[69]
Colorimetric ELISA	0.05 – 50	20	[41,70,71]
Single-particle counting ^k)^	0.017 – 170	17	[72]
Immuno-PCR	0.01 – 100	10	[42]
Electrochemical (quantum dots) ^l)^	0.01 – 80	3.3	[73]
Electrochemical (gold nanoparticle) ^m)^	0.01 – 200	1.5	[74]
Electrochemical (carbon film) ^n)^	0.005 – 50	1	[75,76]
Surface-enhanced Raman scattering ^o)^	0.001 – 0.1	1	[77]
Nanowire sensor array ^p)^	0.001 – 1	0.1	[78]
ILPCR	0.000013 – 13	0.013	this work

^a)^ Only assays performed using human or animal serum and reporting both a dynamic range and *LOD* were included.

^b)^ For the listed assays, the *LOD* is generally defined as the lowest CEA concentration on the dose–response curve ≤ to the blank minus 3-times the standard deviation of the blank (see the individual references for details).

^c)^ Flow injection chemiluminescence immunoassay using a CEA-immobilized immunoaffinity column to capture free HRP-anti-CEA antibodies remaining after incubation with CEA-containing serum.

^d)^ Sandwich immunoassay using capture antibodies immobilized on microarrays based upon the self-assembly of DNA–protein conjugates. CEA is quantified using the fluorescence signal generated from fluorophores conjugated to the (secondary) antibody.

^e)^ Sandwich immunoassay where time-resolved fluorescence emission from a europium-labeled secondary antibody is used to quantify CEA immobilized by a capture antibody.

^f)^ Immunoassay where a electrochemiluminescence signal is generated when CEA labeled with ruthenium (II) binds to capture antibodies immobilized on the surface of an electrode in a competitive assay with unlabeled CEA in serum.

^g)^ Sandwich immunoassay where inductively-coupled plasma (ICP) mass spectrometry (MS) is used to detect CEA from the spectral signal generated by europium-conjugated secondary antibodies bound to CEA immobilized by capture antibodies.

^h)^ Amperometric detection of CEA binding using an immunosensor based on the conjugation of CEA capture antibodies to Au-TiO_2_ hybrid nanocomposite films.

^i)^ Immunoassay using microchip electrophoresis-based detection of free and CEA-bound antibodies labeled with fluorescent tags.

^j)^ Immunoassay in which ICPMS is used to detect catalytic silver deposition initiated by CEA binding to capture antibodies immobilized on gold tags.

^k)^ Single-particle counting of laser-induced photon bursting generated when gold nanoparticles containing CEA bound to conjugated capture antibodies pass through a 1 fL flow cell.

^l)^ Sandwich immunoassay where CEA is detected from the voltammetric stripping pattern that results when metal ions are released from reverse-micelles conjugated to the secondary antibody.

^m)^ Sandwich immunoassay where CEA is detected from the electrochemical signal generated by horseradish peroxidase upon its release from hollow nanogold microspheres conjugated to the secondary antibody.

^n)^ Immunoassay in which an electrochemical signal is detected when CEA binds to capture antibodies conjugated to gold-coated magnetic core-shell nanoparticles immobilized on a carbon-paste electrode.

^o)^ Immunoassay in which surface-enhanced Raman scattering intensity is used to detect CEA bound to capture antibodies conjugated to hollow gold nanosphere magnetic particles.

^p)^ Immunoassay where a conductive signal is generated when CEA binds to capture antibodies immobilized on silicon nanowires fabricated into field-effect transistor sensors.

### ILPCR assay for p24 in buffer

**Figure 6:**
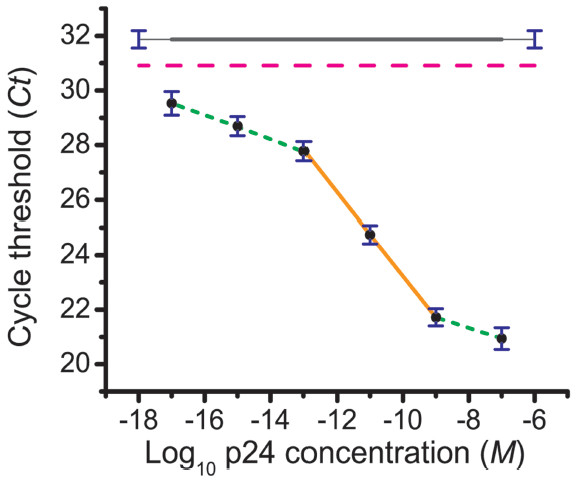
**Quantitative ILPCR dose–response curve for p24 added to PBS buffer.** A 100-μl sample volume (antigen diluted in PBS) was used for all concentrations. The black circles are the average of three replicate *Ct* measurements over a concentration range of 10^-7^ to 10^-17^ M p24; the standard deviations of the *Ct* values are shown as blue vertical bars. In order to be visible, the standard deviations were multiplied by the following values: 10^-7^ M (3x), 10^-9^ M (1x), 10^-11^ M (5x), 10^-13^ M (5x), 10^-15^ M (7x), and 10^-17^ M (1x). The remaining symbols and colors have the same designations as given in Figure 4.

A titration series was prepared by adding recombinant HIV-1 p24 to PBS buffer. The mean *Ct* value and the standard deviation of the calibration standards, the blank, and the controls were calculated using three replicate measurements from the qPCR analysis. The result of this assay is shown in Figure 6. This dose–response curve showed a primary linear region extending from 10^-9^ to 10^-13^ M with a slope of −1.5 Δ*Ct* per log change in concentration, followed by a secondary linear region with a reduced slope of −0.5 Δ*Ct* per log change in concentration that extended from 10^-13^ to 10^-17^ M. Both linear regions exhibited a dynamic range of 4 orders of magnitude. The *LOD*, taken from the primary linear region, was 10^-13^ M, which corresponds to 2.4 pg/ml of p24 or ~6 million molecules of p24 in a 100-μL sample. There are ~3,000 p24 molecules per HIV-1 virion particle [47]; thus, the *LOD* can be restated as 20,000 virions/ml.

The *MDC*, taken from the secondary linear region of the dose–response curve, was at least 10^-17^ M, which corresponds to 0.24 fg/ml of p24 or ~600 molecules of p24 per 100-μl sample. Within the limits imposed by Poisson statistics this is sufficient to detect 2 virions/mL, which is 500-times more sensitive than the best ELISA assays for p24 [48,49]. More important in the clinical management of HIV-1 patients is a viral load in the range of 500 to 1,000 virions/ml. If a patient’s viral load rises significantly above this level failure of antiretroviral therapy is indicated necessitating a change in the treatment protocol [50]. Accordingly, measurement precision in this concentration range is critical. The secondary linear region of the dose–response curve was fit to a linear regression and the upper and lower 95 % confidence limits were calculated. Based upon these results, a p24 concentration corresponding to 500 virions/ml (95 % confidence limit of 341 to 719 virions/ml) can be distinguished from a p24 concentration corresponding to 1,200 virions/ml (95 % confidence limit of 820 to 1,725 virions/ml), which is sufficient for clinical use. It remains to be seen if this precision is realized in actual clinical serum samples that require the disruption of the virion particles by acid or heat treatment to release the p24 core protein [49,51].

We used the p24 ILPCR assay to explore an alternate method for deactivating the DNase I instead of heating the enzyme solution at 80°C for 10 min. Four replicate measurements were performed using the same concentration of p24 in each well. All wells were treated identically through the DNase I digestion step, which was carried out at 37°C for 20 min. Two of the wells were heated at 80°C for 10 min to thermally denature the enzyme, while this step was skipped for the remaining two wells. All four wells were then washed 5 times with 300 μL of *buffer B*. Finally, the liposome detection reagent was lysed by the addition of 100 μL of *lysis buffer* per well, followed by incubation at RT for 20 min on a plate shaker at 600 rpm. All four wells yielded statistically identical *Ct* values for concentrations of 10^-9^ M p24 [20.59 ± 0.51(heat) and 20.12 ± 0.24 (no heat), *p* < 0.05] and 10^-13^ M p24 [27.61 ± 0.41 (heat) and 27.32 ± 0.44 (no heat), *p* < 0.05]. Thus, heat denaturation was not required to neutralize enzyme activity and heating did not disrupt the liposomes or antigen–antibody binding. Washing the wells 5 times with buffer following the DNase I digestion step appeared to remove almost all of the DNase I and the lysis buffer likely had sufficient detergent (10 mM Triton X-100) to denature any remaining enzyme, preventing enzymatic digestion of the amplicons released from the liposomes or the carry-over of active enzyme to the qPCR step. Accordingly, the DNase I heat-deactivation step of the ILPCR assay can be omitted so long as the plate wells are washed thoroughly prior to rupturing the liposomes.

### ILPCR assay controls

The ILPCR assay for CEA included all of the normal qPCR controls; specifically false-positive controls for the lysis buffer, water, the PCR reaction mixture, including the primers and probes, and a no-template control. The assay blank (all of the assay components accept the antigen) served as a control for immunoliposome nonspecific binding. For use in a clinical setting the ILPCR assay will require additional controls. A false-positive result could arise from the failure of the DNase I treatment to digest all non-encapsulated nucleic acid. A non-encapsulated probe, an 89 base-pair reporter derived from the rat GRIP1 sequence, was used for this purpose. A false-negative result could arise from the failure of the detergent to rupture the immunoliposomes, the presence of Taq polymerase inhibitors, or a failure of one of the PCR reagents. A false-negative control was created by encapsulating a third probe, an 81 base-pair reporter derived from the TMV 126 kDa coat protein sequence, inside liposomes where the DSPE-PEG(2000)Biotin was replaced with non-binding DSPE-mPEG(2000). Both controls are added to the assay wells of the microtiter plate immediately prior to the DNase I digestion step.

**Figure 7:**
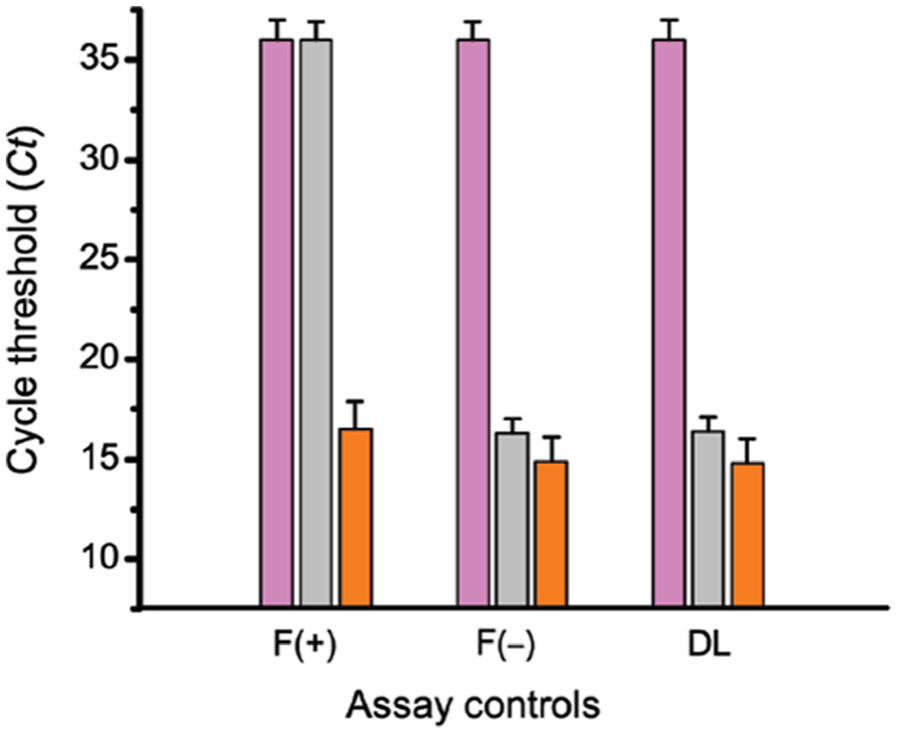
**Performance of the ILPCR assay controls.** F(+): the false-positive control, which is the non-encapsulated GRIP1 reporter; F(―): the false-negative control, which is the TMV reporter encapsulated inside liposomes where the DSPE-PEG(2000)Biotin is replaced with non-binding DSPE-mPEG(2000); DL: the detection liposomes, which contain the β_2_-microglobin reporter encapsulated inside liposomes containing 0.5 mol% DSPE-PEG(2000)Biotin. Magenta columns: *Ct* values obtained when DNase I digestion was performed after rupture of the liposomes. Gray columns: *Ct* values obtained when DNase I digestion, with subsequent heat-deactivation of the enzyme, was performed prior to rupture of the liposomes (normal assay conditions). Orange columns: *Ct* values obtained in the absence of a DNase I digestion step. Measurements were performed using a Bio-Rad model CFX96 real-time PCR system.

The behavior of the false-positive control [F(+)], the false-negative control [F(−)], and the detection liposomes (DL) under various treatment conditions were determined from three multiplex qPCR assays whose results are shown in Figure 7. The concentration of the liposomes and the unencapsulated false-positive control were adjusted to yield equal concentrations of all three reporters. The magenta columns were the *Ct* values obtained when the DNase I digestion step was performed after the rupture of the liposomes. As expected, all *Ct* values were equivalent to the non-template control (*Ct* ≥35). The grey columns were the *Ct* values obtained when DNase I digestion, with subsequent heat-deactivation of the enzyme, was performed prior to the rupture of the liposomes (normal assay conditions). The reporters for the false-negative control and the detection liposomes exhibited a *Ct* of ~15, indicating that the encapsulated reporters were amplified, while the unencapsulated reporter for the false-positive control was not (*Ct* > 35). Finally, the orange columns were the *Ct* values obtained when the DNase I digestion step was omitted, which resulted in all three reporters being amplified (*Ct* values of 15 to 16.5).

**Figure 8:**
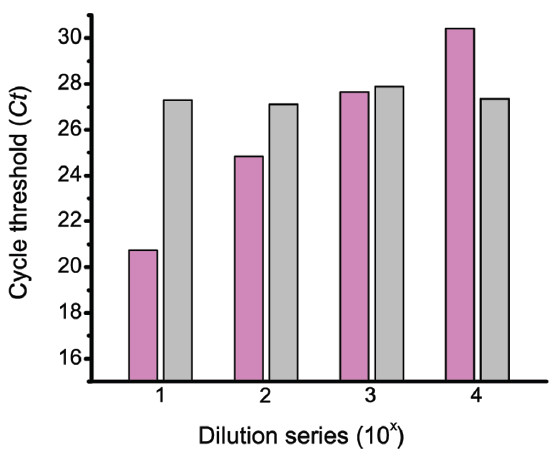
**Performance of a multiplex liposome assay.** The result of a multiplex liposome assay containing a constant concentration of false-negative control liposomes in the presence of a 10,000-fold dilution series of detection liposomes. Magenta columns: *Ct* values of the detection liposomes (β_2_microglobin). Grey columns: *Ct* values of the false-negative control liposomes (TMV). The amplification of the TMV reporter was independent of the concentration of the detection liposomes.

**Figure 9:**
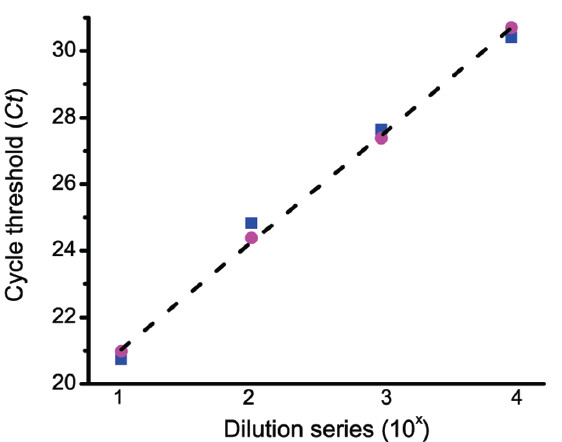
**False-negative control liposomes used as an internal exogenous control.** The filled blue squares are the *Ct* values of the detection liposomes taken from Figure 8. The filled magenta circles are the corrected *Ct* values of the detection liposomes, which were determined from the expression *Ct*_*i*_ x [C*t_*i*_/∑C*t_*i*_], where *Ct*_*i*_ is the cycle threshold value of the detection liposomes from microplate well *i*, C*t_*i*_ is the cycle threshold value of the false-negative control liposomes from microplate well *i*, and ∑C*t_*i*_ is the sum of the cycle threshold values of the false-negative control liposomes from all of the microplate wells. The dashed grey line is the linear regression fit to the corrected *Ct* values of the detection liposomes.

Figure 8 shows the performance of four multiplex qPCR assays containing a 10,000-fold dilution series of the detection liposomes (magenta columns) in the presence of a constant concentration of the false-negative control liposomes (grey columns). The *Ct* values for the TMV reporter encapsulated inside the false-negative control were independent of the *Ct* values of the β_2_-microglobin reporter encapsulated inside the detection liposomes. Because an equal concentration of the false-negative control liposomes was added to each plate well, they can also act as an internal exogenous control [52]. The *Ct* values from the dilution series of the β_2_-microglobin reporter in Figure 8 were re-plotted (blue squares) in Figure 9. A linear fit of these *Ct* values (not shown) yielded a linear correlation coefficient of 0.995, with a standard deviation of 0.504 and *p* < 0.00496. The β_2_-microglobin *Ct* values where then normalized (pink circles) using the false-negative reporter *Ct* values as an internal exogenous standard as described in the legend of Figure 9. A linear fit of these normalized Ct values (dotted line) yielded a linear correlation coefficient of 0.999, with a SD of 0.134 and *p* < 0.000344.

## Conclusions

The ILPCR assay described here was designed to mitigate the two major shortcomings of IPCR, specifically the difficulty in preparing the antibody-DNA conjugates and the difficulty in controlling DNA contamination during the assay. In contrast to conventional IPCR, chemical coupling of the reporter DNA to an antibody to form a conjugate is not required. The reporter DNA and biotin-labeled PEG phospholipid conjugates spontaneously incorporate into the liposomes as they form, thus greatly simplifying the preparation and purification of the detection reagent. Also, the purification of the detection reagent is not critical as any remaining unencapsulated reporter will be degraded during the DNase I digestion step of the assay. More importantly, encapsulation of the reporter DNA inside the liposomes allows contaminating nonspecific DNA in the assay medium to be degraded with DNase I prior to quantification of the encapsulated reporter by qPCR. This unique DNase I digestion strategy also eliminates genomic DNA contamination in the test specimens that could compete with reporter amplification by nonspecific hybridization of the primers [53]. This capability, not possible with IPCR, simplifies specimen preparation and significantly reduces the noise level in the negative assay controls, thus reducing the stringency required to perform the assay. This makes the ILPCR assay format amenable to personnel without extensive experience in PCR techniques or access to PCR-compliant laboratory facilities. The ability to encapsulate multiple reporters per liposome leads to a “pre-amplification” factor, which increases the sensitivity of the assay, and helps overcome matrix effects, including the effect of polymerase inhibitors frequently present in biological specimens [54]. This further reduces the need for extensive sample processing or dilution.

Each liposome has ~800 biotin molecules exposed on its outer bilayer surface, which increases the sensitivity of the assay by increasing the avidity of the detection reagent [55,56]. This can be seen in the dose–response curve of Figure 3. The data from 10^-9^ M to 10^-17^ M was fit to a four parameter logistic model [57] to estimate the effective dissociation constant from the binding curve, which was determined to be ~10^-13^ M. This apparent *K*was lower than the actual *K*of 4 x 10^-11^ M measured for the capture and detection antibodies [38], indicating that the liposomes likely exhibit multivalent binding where one liposome binds to multiple immobilized antigens simultaneously, thereby increasing the sensitivity of the assay.

The very low detection limits that can be achieved with the ILPCR assay make it compatible with high-throughput qPCR-based microarray platforms [58,59]. The CEA assay described here is sufficiently sensitive to yield a detection limit of ~1 pg/mL for a serum volume of 1 μL or ~1 ng/mL for a serum volume of 1 nL. Further, the generic biotin-labeled liposomes can be coupled through a NeutrAvidin bridge to a multitude of biotin-labeled probes, including carbohydrates, antibodies, aptamers, proteins, DNA, RNA, and peptide nucleic acids. Thus, it is envisioned that ILPCR could form the foundation of a qPCR-based high-throughput ultrasensitive quantitative assay system where genomic, epigenetic, proteomic, glycomic, and immunologic assays can be carried out simultaneously on a single positionally-encoded microarray chip or plate. Such heterogeneous assay platforms may be the key technologic advance in linking the remarkable growth in our knowledge of the molecular pathology of disease to meaningful clinical correlations. Accordingly, we believe that ILPCR holds great promise as a clinical diagnostic assay method. See [79-83] for references related to material in additional file 1: Supplementary information.

## Competing interests

TJO and JTM are listed as inventors on two United States patents, numbers 7,582,430 B2 (1 September, 2009) and 7,662,568 B2 (16 February, 2010) covering the immunoliposome detection reagent and assay technology described in this paper. The assignee on these patents is the United States of America as represented by the Secretary of the Army. There was no support or involvement, financial or otherwise, by any commercial entity in the work described in this paper.

## Authors’ contributions

JH planned and carried out all of the ILPCR CEA and p24 assay development and optimization work, prepared the liposome detection reagents, assisted in the characterization of the liposome detection reagents, and in the preparation of the manuscript. DLE performed the ILPCR assay control experiments. TJO and JTM contributed extensive technical consultation and expertise on the design of the ILPCR assay format. JTM assisted in the characterization of the liposome detection reagents and in the prepared of the manuscript. All authors read and approved the final submitted version of the manuscript and all supporting material.

## Supplementary Material

Additional file 1 Supplementary information: (1) Preparation of DNA reporters, (2) Reporters, primers, and probes, and (3) Pre-column nuclease digestion of reporters. Click here for file
