# Supplementary material for: Immunoliposome-PCR: a generic ultrasensitive quantitative antigen detection system
Source: J Nanobiotechnology. 2012 Jun 22;10:26. doi: 10.1186/1477-3155-10-26 (PMC3466442; doi:10.1186/1477-3155-10-26)
Supplement: Additional file 1 — Supplementary information: (1) Preparation of DNA reporters, (2) Reporters, primers, and probes, and (3) Pre-column nuclease digestion of reporters. [file 1477-3155-10-26-S1.pdf]

ADDITIONAL FILE 1

Immunoliposome-PCR: a generic ultrasensitive quantitative antigen detection system

Junkun He[1,2], David L. Evers[1,2], Timothy J. O'Leary[1], and Jeffrey T. Mason[1,2*]

[1]Biomedical Laboratory Research and Development Service, Veterans Health Administration, Washington, DC, USA. [2]Armed Forces Institute of Pathology, Rockville, MD, USA

*Corresponding author: Jeffrey T. Mason, PhD, Washington DC VA Medical Center, Research Building, Room GF-147, 50 Irving Street, N.W., Washington, DC 20422. Phone (301-318-5626), Fax (202-518-4645), Email (jeffrey.mason.afip@gmail.com)

Email addresses:

JH: junkunhe@yahoo.com; DLE: eversDL@comcast.net; TJO: timothy.oleary@va.gov;

JTM: jeffrey.mason.afip@gmail.com

Additional file 1 contains the following information:

Preparation of DNA reporters

Reporters, primers, and probes

Pre-column nuclease digestion of reporters

References

Preparation of the β_2-myoglobin reporter

Materials

PCR primers were purchased from Integrated DNA Technologies (Coralville, IW) and AmpliTaq Gold DNA polymerase, Taqman universal PCR mastermix, and the PCR Taqman probes were purchased from Applied Biosystems (Carlsbad, CA). The TOPO TA cloning kit with pCR2.1-TOPO T/A plasmid vector and One-Shot *E. coli* was purchased from Invitrogen (Carlsbad, CA). The TRizol Plus RNA purification kit, SuperScript First-Strand Synthesis System for RT-PCR and 10x PCR buffer were also purchased from Invitrogen. The Plasmid DNA Mini-Prep kit and QIAquick PCR purification kit were purchased from Qiagen (Valencia, CA). Sodium acetate solution (3 M), glycogen, and absolute ethanol were purchased from Sigma-Aldrich (St. Louis, MO). HeLa cells were obtained from the ATCC (Manassas, VA).

Preparation of the β_2-microglobin amplicon

HeLa cells (~1 x 10^7 cells) in PBS, pH 7.4, were pelleted by centrifugation at 300 x g for 10 min. The total RNA was extracted from the cell pellet using the TRizol Plus RNA Purification kit. The isolated RNA was then converted to cDNA with the SuperScript RT-PCR First-Strand Synthesis System using random primers.

The β_2-microglobin amplicon was amplified from the cDNA derived from the HeLa cells by PCR using the β_2M-246F and β_2M-330R primers whose sequences are given in the following section. All primer and probe design was performed using Taqman Probe & Primer Design software (Applied Biosystems). The presence of the amplicon was confirmed by agarose gel electrophoresis. If more than one band was present the correct amplicon was isolated by extraction from the appropriate gel band.

The amplicon was then cloned into the pCR2.1-TOPO T/A plasmid vector, which was used to transform One-Shot chemically-competent *E.coli* using the TOPO TA cloning kit. The *E. coli* were then incubated overnight at 37°C on appropriate LB plates (Ampicillian and X-gal). Ten white or light blue colonies were isolated and cultured overnight in LB medium containing 50 µg/mL Ampicillin.

The positive transformants were analyzed by PCR using forward or reverse M13 primer plus the β_2M-246F or β_2M-330R primer for the β_2-microglobin insert. If desired, a restriction analysis can be performed in parallel. Once the correct clone was identified, a stock solution was prepared by culturing the colony in 1-2 mL of LB containing 50 μg/mL of Ampicillin. The culture was allowed to reach stationary phase, after which 1mL of the culture was combined with 0.15 mL of sterile glycerol, transferred to a cryovial, and stored at -80°C.

A stock solution of the amplicon was prepared by extracting the plasmid DNA using the Plasmid Mini-Prep kit. A 328 bp DNA fragment containing the β_2-microglobin transcript was amplified from the recombinant plasmid by using M13 forward and reverse primers. This was done to ensure that only the β_2-microglobin reporter was amplified in the final PCR step. PCR amplification was carried out using AmpliTaq Gold DNA polymerase, Taqman universal PCR mastermix, and the standard protocol of 29 cycles as shown below. Following PCR, the presence of the 328 bp fragment was confirmed by agarose gel electrophoresis.

Cycle number	Denature	Anneal	Extend
1-25	95°C for 60 s	55°C for 1 min	72°C for 3 min
26			72°C for 3 min

The 84-bp DNA reporter was generated by amplifying the 328-bp amplicon with the β_2M-246F and β_2M-330R primer set (15 μM each) using the same PCR protocol described above. Following PCR, the presence of the 84-bp reporter was confirmed by agarose gel electrophoresis. The reporter was purified using a QIAquick PCR purification kit and then precipitated at -20°C overnight by adding 1/10 (v/v) of 3M sodium acetate, pH 5.2, and three volumes of absolute ethanol containing glycogen (1 ng/mL) as a carrier. The DNA solution was centrifuged at 16,000 x g for 25 min at 23°C. The pellet was washed with 70% ethanol and dried under a stream of nitrogen.

The reporter was then dissolved in 500 µL of 10 mM Tris-HCl, pH 7.4, and the reporter concentration was determined by measuring the absorbance at 260 nm. For the β_2-microglobin reporter an absorbance of 1.0 at 260 nm corresponds to a concentration of 50 µg/mL. The reporter was diluted to 667 µg/mL and stored at -80°C. The reporter is stable for ~ 2years [1]. Other reporters can be prepared as described above by using appropriate primers. Alternately, reporters can be ordered from commercial sources including Invitrogen, Integrated DNA Technologies, and GenScript (Piscataway, NJ).

Reporters, primers, and probes

1. **Sequence, primers, and probe for the β_2-myoglobin reporter** [2]:

   Source: Human beta 2-microglobin gene sequence [GenBank: NG_012920.1]

   Reporter: (length: 84 bp): `TGA CTT TGT CAC AGC CCA AGA TAG TAA GTG GGA TCG AGA CAT GTA AGC AGC ATC ATG GAG GTT TGA AGA TGC CGC ATT TGG ATT`

   Forward Primer (β_2M-246F): `5'-TGA CTT TGT CAC AGC CCA AGA TA-3'`

   Reverse Primer (β_2M-330R): `5'-AAT CCA AAT GCG GCA TCT TC-3'`

   Probe: `5'-[VIC] TGA TGC TGC TTA CAT GTC TCG ATC CCA [TAMRA]-3'`

   Fluorophores: TAMRA (quencher); VIC (absorbance λ_{max} = 538 nm, emission λ_{max} = 554 nm)

   Concentration: A_{260} x 55.8 nmol/mL and A_{260} x 1,458 µg/mL (includes the assay dilution factor as described in the Methods section of the parent publication)

2. **Sequence, primers, and probe for the TMV 126 kDa coat protein reporter** [3]:

   Source: cDNA transcript derived from the tobacco mosaic virus 126 kDa coat protein linear RNA sequence [GenBank: NC_001367.1]

Reporter: (length: 81bp) AAT CCG ACA TGG CGA AAC TCA GAA CTC TGC GCA GAC TGC TTC GAA ACG GAG AAC CGC ATG TCA GTA GCG CAA AGG TTG TAA

Forward Primer: 5'-GAC ATC AAG AAG GTG GTG AAG CAG -3'

Reverse Primer: 5'-GTT GAA GTC ACA GGA CAC AAC CTG-3'

Probe: 5'-[6-FAM] TCA AGG GCA TCC TGG GCT ACA CTG A [TAMRA]-3'

Fluorophores: TAMRA (quencher); 6-FAM (absorbance λ_{max} = 494 nm, emission λ_{max} = 518 nm)

Concentration: A_{260} x 57.9 nmol/mL and A_{260} x 1,452 µg/mL (includes the assay dilution factor as described in the Methods section of the parent publication)

3. **Sequence, primers, and probe for the GRIP1 reporter** [4]:

Source: cDNA transcript derived from the Norway rat glutamate receptor-interacting protein 1 linear RNA sequence [GenBank: NM_032069.1]

Reporter: (length: 89 bp) GCA CCA AAG CAG ACA CAA GTT AAA GAA AGA TAT TCT CCT CAT GGA GCA GAG GAT GAA TCT TGG AGA AGC ATG GGC TTC AAC AAT GAC AA

Forward Primer: 5'-GCA CCA AAG CAG ACA CAA GT-3'

Reverse Primer: 5'-ATG GGC TTC AAC AAT GAC AA-3'

Probe: 5'-[TET]-TCA TGG AGC AGA GGA TGA ATC TTG GA-[TAMRA]-3'

Fluorophores: TAMRA (quencher); TET (absorbance λ_{max} = 521 nm, emission λ_{max} = 536 nm)

Concentration: A_{260} x 52.7 nmol/mL and A_{260} x 1,542 µg/mL (includes the assay dilution factor as described in the Methods section of the parent publication)

Pre-column nuclease digestion of reporters

Deoxyribonuclease I (DNase I) from bovine pancreas (~2,000 Kunitz units/mg protein), exonuclease III from *Escherichia coli* (~150 Kunitz units/µL), and DEAE-Sepharose CL-6B were obtained from Sigma-Aldrich (St. Louis, MO). The exonuclease III is supplied in a buffer consisting of 5 mM potassium phosphate, pH 6.5, 200 mM KCl, 0.05 mM EDTA, 5 mM 2-mercaptoethanol, 200 µg/mL bovine serum albumin, 50% (v/v) glycerol. DNase I (15,000 Kunitz units, 7.5 mg) and exonuclease III (10 µL) were dissolved in 240 µL of digestion buffer consisting of 50 mM $CaCl_2$, 50 mM $MgCl_2$, 20 mM HEPES, pH 7.8. This mixture was added to 1 mL of the crude liposome preparation (25 mg total lipid) and the solution was stirred for 3 h at 37°C [5]. The nucleases were then inactivated by heating the liposome preparation for 10 min at 80°C. The degraded reporter DNA and nucleases were removed by ion-exchange gel filtration on DEAE-Sepharose CL-6B (0.5 mL of gel/mg total lipid) by eluting the column with 20 mM HEPES, pH 7.5, 145 mM NaCl.

References

1. Mason JT, Xu L, Sheng ZM, He J, O'Leary TJ: **Liposome polymerase chain reaction assay for the sub-attomolar detection of cholera toxin and botulinum neurotoxin type A**. *Nature Protocols* 2006, **4**: 2003-2011.

2. Güssow D, Rein R, Ginjaar I, Hochstenbach F, Seeman G, Kottman A, Ploegh HL: **The human beta 2-microglobin gene: primary structure and definition of the transcriptional unit**. *J Immunol* 1987, **139**: 3132-3138.

3. Goelet P, Lomonossoff GP, Butler PJG, Akam ME, Gait MJ, Karn J: **Nucleotide sequence of tobacco mosaic virus RNA**. *Proc Natl Acad Sci USA* 1982, **79**: 5818-5822.

4. Dong H, O'Brien RJ, Fung ET, Lanahan AA, Worley PF, Huganir RL: **GRIP: a synaptic PDZ domain-containing protein that interacts with AMPA receptors.** *Nature* 1997, **386:** 279 –284.

5. Monnard P-A, Oberholzer T, Luisi PL: **Entrapment of nucleic acids in liposomes**. *Biochim Biophys Acta* 1997, **1329**: 39-50.
